# P025 Painful feet: an unusual presentation of antiphospholipid syndrome

**DOI:** 10.1093/rap/rkag113.055

**Published:** 2026-11-03

**Authors:** Daniel Coulter, Ashley Elliott, Natalie McKee

**Affiliations:** Ulster Hospital Dundonald, Belfast, United Kingdom; Musgrave Park Hospital, Belfast, United Kingdom; Musgrave Park Hospital, Belfast, United Kingdom

## Abstract

**Introduction:**

Antiphospholipid syndrome (APS) has traditionally been diagnosed by the presence of both a clinical thrombotic event or pregnancy complication alongside persistently positive antiphospholipid antibodies (aPL). Recent updates to the American College of Rheumatology (ACR)/ European League Against Rheumatism (EULAR) diagnostic criteria have broadened the clinical criteria to include microvascular thrombotic events such as livedoid vasculopathy, meaning APS can be diagnosed without a macrovascular or obstetric event. We present an unusual case of APS whose initial clinical features were painful feet with an associated rash.

**Case description:**

A 62-year-old male was referred to rheumatology with a 6-month history of burning pain in both feet. He had a past medical history of hypertension managed with amlodipine and had a 30-pack-year smoking history. A couple of months after the pain started he developed a purpuric rash on his feet and legs and reported the toes in his left foot turned intermittently cold and dusky. When seen in rheumatology outpatients in August 2025 painful erythematous nodules were noted on his hands and knees and a purpuric rash was present on his legs. Aside from occasional night sweats he was otherwise systemically well and had no history of thrombotic disease. Ankle-brachial pressure index was normal as were screening bloods carried out in primary care including full blood count, kidney function, complement levels, rheumatoid factor and a connective tissue disease screen. At this stage the working diagnosis was a potential cryoglobulinaemia and further tests including a CT Chest Abdomen and Pelvis and APS screen were requested with a plan for further outpatient review. Cryoglobulin levels returned as normal however all three of his aPL antibodies came back strongly positive with a Cardiolipin IgG of 432U/ml, Beta-2 glycoprotein IgG of 2887U/ml and lupus anticoagulant of 56.1s. The decision was made to admit the patient for inpatient investigations including a skin biopsy which showed thrombotic occlusions of the small arterioles of the deeper dermis with fibrin plugs in keeping with APS. He was commenced on warfarin with a target INR of 2.5 alongside long-term hydroxychloroquine and a reducing course of prednisolone to treat any associated vasculopathy. All three aPL remained positive at repeat testing in three months and the diagnosis of triple-positive APS was secured.

**Discussion:**

This case presented a number of diagnostic and management challenges. His skin biopsy fulfilled the clinical criteria as a livedoid vasculopathy and given the strength of the positive initial aPL antibodies the decision was made to commence treatment with warfarin before awaiting the repeat antibodies. This is a somewhat unusual clinical dilemma as usually patients present with a macrovascular event that warrants treatment initially regardless of aPL status, giving clinicians time to await the repeat antibody results before deciding on a long-term anticoagulant strategy. He had none of the classical thrombotic manifestations of APS.

During his admission a CT angiogram lower limbs was performed showing severe stenosis of his left distal superficial femoral artery (SFA) which was initially medically managed due to the lack of critical limb ischaemia. A few months after commencement of warfarin the patient developed a persistently dusky left second toe so the decision was made to revascularise the leg with balloon angioplasty to the distal left SFA. After this alongside his warfarin he received three months of dual anti-platelet therapy with aspirin and clopidogrel. This greatly improved his pain and skin changes however his livedoid rash and pain returned about one month after discontinuing anti-platelet therapy, despite having a therapeutic INR. The patient had dual pathology with peripheral vascular disease that was made worse by the APS. Repeat imaging of his lower limb vasculature demonstrated no critical stenosis and so this was treated as a recurrent microthrombotic event. Discussions were had with the haematology team whether to increase his INR target to 3.5 or add in an anti-platelet along with warfarin and keep INR target at 2.5. The latter management option was chosen and his symptoms have largely settled, though an ongoing rash in keeping with livedo racemosa remains.

**Key learning points:**

• This case raises a number of learning points. Firstly it highlights the enhanced understanding in recent years of the clinical manifestations of APS that are not macrovascular venous or arterial thrombosis which can still contribute to the diagnosis. At initial rheumatology assessment the patient was displaying a livedo racemosa rash alongside livedoid vasculopathic lesions. These findings are often difficult to detect clinically, especially in the absence of any thrombotic events which would increase our suspicion of APS. Furthermore clinical appearance alone of these findings is not sufficient to fulfil the clinical component of the ACR/EULAR criteria, instead it needs to be confirmed with a biopsy. This demonstrates the potential utility of skin biopsy in diagnosis and is reflected in the British Journal of Haematology guidelines on APS which states the presence of a livedoid rash should trigger laboratory screening for aPL even without any history of thrombosis.

• This case also reinforces the need for close collaboration with colleagues in other specialties with dermatology, haematology and vascular surgery all playing a role in this case alongside the rheumatology team. The dermatology team aided prompt diagnosis by performing a skin biopsy and haematologist’s expertise was valuable when symptoms returned to guide the complex decision making around managing recurrent microthrombosis. The need for surgical intervention highlighted how both APS and traditional cardiovascular risk factors such as smoking and hypertension can coexist to increase your risk of macrothrombotic events. Whilst making complex decisions regarding anticoagulant and potentially immunosuppressive strategies it is easy to forget to reinforce basic cardiovascular risk reduction therapies such as smoking cessation and strict control of blood pressure, lipids and blood sugar. This patient has engaged in measures to reduce his atheroma risk, having stopped smoking and is compliant with anti-hypertensive and anti-lipid treatments.

